# The Targeted Regulation of BDUbc and BDSKL1 Enhances Resistance to Blight in *Bambusa pervariabilis* × *Dendrocalamopsis grandis*

**DOI:** 10.3390/ijms25010569

**Published:** 2024-01-01

**Authors:** Peng Yan, Yisi Wang, Cailin Yu, Jingmei Piao, Shuying Li, Yinggao Liu, Shujiang Li

**Affiliations:** 1 College of Forestry, Sichuan Agricultural University, Chengdu 611130, China; 2023104008@stu.sicau.edu.cn (P.Y.); 202101498@stu.sicau.edu.cn (Y.W.); 202101511@stu.sicau.edu.cn (C.Y.); 202101495@stu.sicau.edu.cn (J.P.); 72111@sicau.edu.cn (S.L.); 11468@sicau.edu.cn (Y.L.); 2 National Forestry and Grassland Administration Key Laboratory of Forest Resources Conservation and Ecological Safety on the Upper Reaches of the Yangtze River, Chengdu 611130, China

**Keywords:** *Bambusa pervariabilis* × *Dendrocalamopsis grandis*, blight, *BDUbc*, *BDSKL1*, overexpression, synergistic enhancement

## Abstract

*Arthrinium phaeospermum* is the major pathogen responsible for the significant stem disease “blight” in *B. pervariabilis* × *D. grandis*. The interacting proteins of the key pathogenic factor *ApCtf1β*, BDUbc and BDSKL1, have previously been obtained by two-hybrid, BiFC, GST pull-down yeast assays. However, the functions of these interacting proteins remain unknown. This study successfully obtained transgenic plants overexpressing *BDUbc*, *BDSKL1*, and *BDUbc* + *BDSKL1* via Agrobacterium-mediated gene overexpression. qRT-PCR analysis revealed significantly increased expression levels of *BDUbc* and *BDSKL1* in the transgenic plants. After infection with the pathogenic spore suspension, the disease incidence and severity index significantly decreased across all three transgenic plants, accompanied by a marked increase in defense enzyme levels. Notably, the co-transformed plant, OE-*BDUbc* + *BDSKL1*, demonstrated the lowest disease incidence and severity index among the transgenic variants. These results not only indicate that *BDUbc* and *BDSKL1* are disease-resistant genes, but also that these two genes may exhibit a synergistic enhancement effect, which further improves the resistance to blight in *Bambusa pervariabilis* × *Dendrocalamopsis grandis*.

## 1. Introduction

Bamboo plants are essential economic resources, and their economic and ecological value has been gradually gaining increased attention, with bamboo forests being referred to as the “second forest”. *Bambusa pervariabilis* × *Dendrocalamopsis grandis* is a superior *B. pervariabilis* × *D. grandis* variety developed through a 12-year breeding program. It is derived from the mother plant *Bambusa pervariabilis*, characterized by its small size, branching abundance, and ease of propagation, and the larger *Bambusa grandis*, serving as the father plant [1]. In addition to exhibiting the advantages of both parent plants, this bamboo variety also shows clear characteristics of growth superiority and ease of asexual reproduction. *B. pervariabilis* × *D. grandis* offers significant economic and societal benefits. It serves as an excellent source of timber and fiber materials, particularly suitable for paper production and related industries. As society continues to grow economically, the applications of bamboo fibers are expanding, making bamboo a stable and promising source of renewable materials with minimal risk in the face of rising costs. Furthermore, *B. pervariabilis* × *D. grandis* is also a high-quality source of bamboo shoots and ornamental bamboo. Bamboo shoots, a completely natural and nutritious green food, are delicious and possess substantial medicinal value. Bamboo wood is extensively used in various fields, such as bamboo weaving, root carving, and the production of bamboo wood composite boards. Additionally, it plays a vital role in promoting the construction of ecological barriers in the Yangtze River basin region [2,3]. While *B. pervariabilis* × *D. grandis* possesses numerous advantages such as strong reproductive capability, easy cultivation, high yield, and versatile applications, its widespread cultivation has faced challenges due to various fungal diseases. These include bamboo wilt disease, blight disease, and scab disease. These fungal diseases have dealt a devastating blow to the *B. pervariabilis* × *D. grandis* industry.

*B. pervariabilis* × *D. grandis* has experienced widespread dieback in recent years, resulting in significant economic losses. It has been confirmed that *A. phaeospermum* is the primary pathogenic fungus responsible for causing blight in *B. pervariabilis* × *D. grandis* [4]. *A. phaeospermum* has a broad host range and strong pathogenicity, capable of infecting 56 different plant species [5,6,7], leading to the wilting and decay of host plants. Research on blight in *B. pervariabilis* × *D. grandis* is still in its early stages, primarily focusing on physiological aspects. This includes studying the disease occurrence patterns, the biological characteristics of the pathogen, toxicity assessment of pathogenic toxin proteins, and the isolation and screening of antagonistic bacteria against the disease [8,9,10]. These studies provide an important molecular biology foundation for further exploring the mechanisms underlying blight occurrence in *B. pervariabilis* × *D. grandis* and for breeding disease-resistant varieties. In our previous research, we conducted a combined transcriptome analysis of *A. phaeospermum* under different culture conditions and the interaction transcriptome between *A. phaeospermum* and *B. pervariabilis* × *D. grandis*. This analysis led to the identification of the key pathogenic gene *ApCtf1β*, which was confirmed to be the critical pathogenic gene responsible for infecting *B. pervariabilis* × *D. grandis* [11]. An analysis of the secondary and tertiary structures of *ApCtf1β* reveals the presence of 18 α helices, 12 β folds, and a zinc ligand in its tertiary structure. The deletion mutant Δ*ApCtf1β* was obtained using PEG-mediated protoplast gene knockout, revealing that *Ctf1β* can suppress the host’s reactive oxygen immune response and interfere with its cell wall formation. Additionally, gene knockout complementation experiments were used to eliminate phenotypic genetic changes induced by protoplast transformation. The pathogenicity index results demonstrate that the virulence of the Δ*ApCtf1β* mutant is lower than that of the wild-type strain. In contrast, the complemented strain’s virulence is restored to wild-type levels, indicating a potential close association between the *ApCtf1β* gene and the virulence of *A. phaeospermum*. The origin of cutinase research lies in exploring the pathogenic mechanisms plant pathogens employ. Pathogens secrete cutinases to break down the cuticle layer on the plant’s surface [12,13], enabling them to invade the plant and impact its growth. The transcriptional activation of cutinase genes in *Fusarium solani* and *Aspergillus nidulans* is mediated by the cutinase transcription factor CTF1 [14]. Currently, there is limited research on the screening and functional analysis of cutinase transcription-factor-interacting target proteins in host plants, particularly in bamboo species. This knowledge gap significantly hinders a deeper understanding of the pathogenic mechanisms of *A. phaeospermum* and the molecular mechanisms underlying the response of *B. pervariabilis* × *D. grandis* to invasion by it. Therefore, utilizing molecular biology techniques to elucidate the functions of cutinase transcription-factor-interacting target proteins in *B. pervariabilis* × *D. grandis* is essential to establishing a foundation for understanding the molecular mechanisms of disease resistance in *B. pervariabilis* × *D. grandis*.

*B. pervariabilis* × *D. grandis* possesses a well-developed epidermal layer. In addition to its roles in water retention and barrier functions, this layer also plays a complex defensive role in promoting overall plant development and regulating interactions between the plant and pathogens [15,16,17]. Cutinase is considered an important enzyme for fungi to penetrate the epidermal layer and infect plants. It plays a crucial role in adhesion to the plant epidermis, penetration of the epidermal layer, and signal generation [18,19]. In the earlier stages of our research, we screened and verified proteins that interact with *ApCtf1β*, namely BDUbc and BDSKL1. Subsequently, we cloned and analyzed the full-length coding sequences (CDS) of *BDUbc* (ubiquitin-conjugating enzyme) and *BDSKL1* (shikimate kinase-like protein 1) in *B. pervariabilis* × *D. grandis*. Multiple sequence alignments and a phylogenetic tree analysis indicated that the obtained *BDUbc* and *BDSKL1* proteins exhibit significant functional similarity to Ubcs and SKL1s identified in the alignment [2].

The ubiquitin–proteasome pathway (UPP) is widely present in eukaryotic organisms. It plays a crucial role in various cellular processes, including maintaining cellular function, regulating the cell cycle, responding to environmental stress, embryonic development, hormone responses, and aging [20,21,22,23,24,25]. The primary role of ubiquitin-conjugating enzymes (E2) is to form a multi-ubiquitin chain in conjunction with ubiquitin-activating enzymes (E1), ubiquitin ligases (E3), and target proteins. This ubiquitination process results in the substrate protein being tagged with ubiquitin and recognized and degraded by the 26S proteasome [20,26], ultimately completing a three-step enzymatic cascade reaction. This process plays a crucial role in maintaining the balance between protein synthesis and degradation within the cell, thereby contributing to maintaining cellular homeostasis and normal functioning [26,27]. Existing research findings indicate that ubiquitin-conjugating enzymes play a role in regulating various aspects of plant growth and development, as well as responses to environmental stress. These include root growth and development [28], photomorphogenesis in plants [29], flowering in plants [30], resistance to salt stress [31], and the balance of plant nutrient metabolism [32]. Furthermore, the expression of plant ubiquitin-conjugating enzyme genes is tissue-specific and subject to regulation by external environmental stressors [31,33,34]. The shikimate pathway is closely associated with various processes in plants, including growth and development, stress resistance, and secondary metabolism [35,36]. This pathway directs carbon from the central metabolic pool to various secondary metabolites involved in plant development, growth, and stress responses. SKL1 is a functionally distinct paralog that has evolved from plant shikimate kinase (SK) genes through repeated evolution. However, SKL1 possesses structural domains that differ from ancestral shikimate kinase, lacking the conserved shikimate binding and catalytic residues [37]. Research has shown that *AtSKL1* plays a significant role in chloroplast development in *Arabidopsis* [38], while in maize, *ZmSKL1* positively regulates drought tolerance against drought stress [39]. However, despite the absence, to date, of research regarding the roles of Ubcs and SKL1s in the biotic stress responses of bamboo plants, inference from studies in plants such as *Arabidopsis*, rice, and barley, among others, suggests that Ubcs and SKL1s play pivotal roles in plant defense against biotic stress [37,38,40,41,42]. Hence, exploring the functions of *BDUbc* and *BDSKL1* is crucial to supporting the resistance of *B. pervariabilis* × *D. grandis* against *A. phaeospermum*.

This study involved a bioinformatics analysis of the amino acid sequences of the key pathogenic factor *ApCtf1β* and its interacting proteins, *BDUbc* and *BDSKL1*, in the host *B. pervariabilis* × *D. grandis*. Through Agrobacterium-mediated gene overexpression, transgenic lines with overexpressed interacting genes were obtained. Ultimately, the physiological and biochemical parameters and pathogenicity differences between pathogen-infected mutant plants and wild-type plants were compared to validate the functions of *BDUbc* and *BDSKL1* proteins. This research aims to elucidate the disease-resistant functions of *BDUbc* and *BDSKL1*, leading to the development of highly disease-resistant transgenic plants. This, in turn, provides a reliable theoretical foundation for studying the pathogenic pathway of *A. phaeospermum* and the molecular resistance mechanisms of *B. pervariabilis* × *D. grandis* to pathogen invasion. It also offers a basis for developing new strategies for the continuous and effective control of blight in forest trees.

## 2. Results

### 2.1. Bioinformatics Analysis of BDUbc and BDSKL1

**Figure 1:**
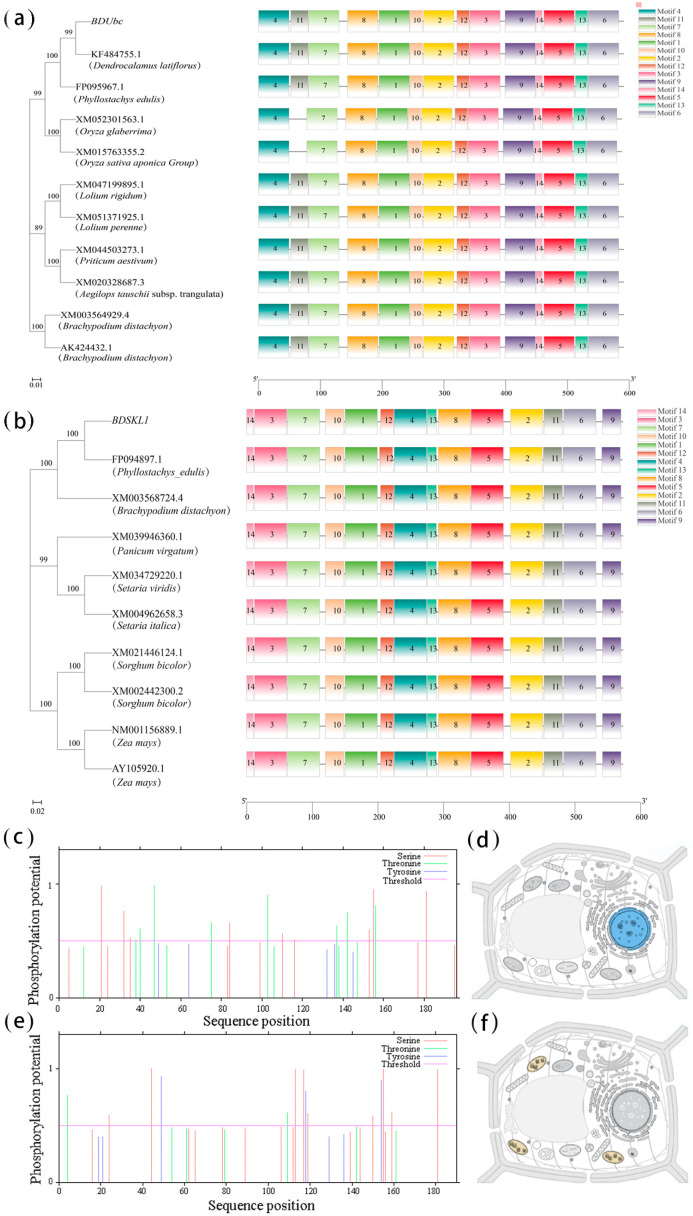
Bioinformatics analysis of *BDUbc* and *BDSKL1*. (**a**) Phylogenetic tree and conserved domain analysis of *BDUbc*. (**b**) Phylogenetic tree and conserved domain analysis of *BDSKL1*. (**c**) Phosphorylation site prediction for *BDUbc*. (**d**) Subcellular localization prediction for *BDUbc* (blue indicates the nucleus). (**e**) Phosphorylation site prediction for *BDSKL1*. (**f**) Subcellular localization prediction for *BDSKL1* (yellow indicates chloroplast).

The phylogenetic tree analysis revealed that *BDUbc* exhibits a closer phylogenetic relationship with *Dendrocalamus latiflorus*, with a homology of 97.45% to *DlUbc* (AGY80454.1). In contrast, *BDSKL1* shows a closer phylogenetic relationship with *Phyllostachys edulis*, with a homology of 91.58% to *PeSKL1* (AIA26163.1) (Figure 1a,b). The structural domain prediction results, as depicted in Figure 1a,b, indicate that *BDUbc* and *BDSKL1* genes are highly conserved, with each gene identified with 14 conservative motifs (motif1-14). Motif1 exhibits the highest confidence score (Figure 1a,b). The NetPhos 3.1 Server prediction results reveal the presence of 17 phosphorylation sites in *BDUbc*, including 9 serine (Ser) phosphorylation sites and 8 threonine (Thr) phosphorylation sites. In *BDSKL1*, there are 14 phosphorylation sites, comprising 9 serine (Ser) phosphorylation sites, 2 threonine (Thr) phosphorylation sites, and 3 tyrosine (Tyr) phosphorylation sites (Figure 1c,e). The PredictNLS online software (https://rostlab.org/owiki/index.php/PredictNLS, 15 August 2023) predicts cellular localization, indicating that *BDUbc* is localized in the nucleus, while *BDSKL1* is localized in the chloroplast (Figure 1d,f).

### 2.2. Construction of Overexpression Vectors

Primer sequences were designed with homologous arms of 15 bp before and after the KpnI restriction endonuclease sites on pCAMBIA1301-35SN and pSuper1300-GFP (Table S1). Using cDNA from the *B. pervariabilis* × *D. grandis* as a template, gene sequences of BDUbc and BDSKL1 were amplified, resulting in fragments of 591 bp and 573 bp, respectively. Recombinant plasmids, namely pCAMBIA1301-35SN-BDUbc, pCAMBIA1301-35SN-BDSKL1, pSuper1300-GFP-BDUbc, and pSuper1300-GFP-BDSKL1, were obtained through homologous recombination. Bacterial liquid PCR analysis confirmed their alignment with the theoretical values, and sequencing validation revealed their positivity without any base mutations (Figure S1a,c). The above recombinant plasmids were transferred into Agrobacterium tumefaciens using the freeze–thaw method. After culturing at 28 °C for 2 d, single colonies were selected for colony PCR validation using BDUbc-F/R and BDSKL1-F/R primers. The fragment sizes matched the theoretical values, and sequencing validation confirmed their positivity without any base mutations (Figure S1b,d).

### 2.3. A. tumefaciens Tumefaciens-Mediated Overexpression in the Genetic Transformation of B. pervariabilis × D. grandis

#### 2.3.1. The Genetic Transformation of *B. pervariabilis* × *D. grandis*

**Figure 2:**
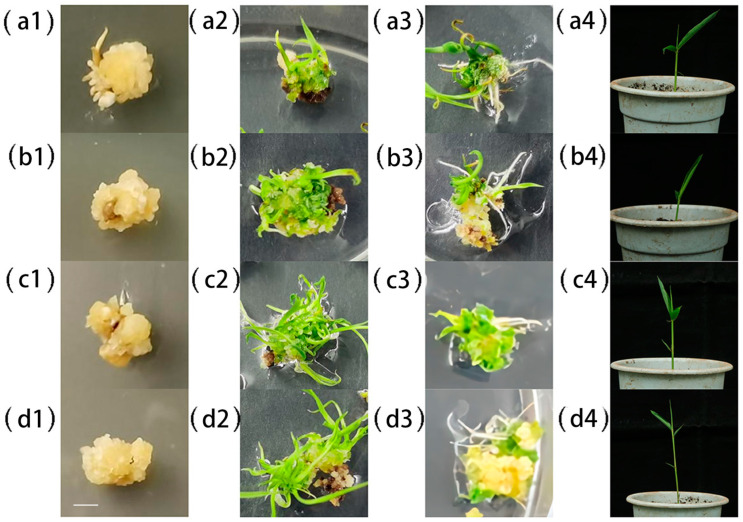
Genetic transformation of *B. pervariabilis* × *D. grandis*. (**a1**–**a4**) Wild-type *B. pervariabilis* × *D. grandis*; (**b1**–**b4**) OE-*BDUbc*; (**c1**–**c4**) OE-*BDSKL1*; (**d1**–**d4**) OE-*BDUbc* + *BDSKL1*; 1: embryogenic callus tissue; 2: shoot differentiation; 3: root differentiation; 4: transgenic plants after transplantation. The white bar indicates 1 cm.

Following the induction, co-culture, differentiation, seedling growth, rooting, hardening, and transplantation stages, transgenic *B. pervariabilis* × *D. grandis* seedlings were obtained, as shown in Figure 2. Embryogenic callus tissues were differentiated into green shoots in a pre-differentiation medium. Later, embryogenic callus tissues were used to separate shoot buds on differentiation medium. After more passages, green shoots were obtained. After rooting culture, the differentiated shoots were transplanted as embryogenic callus-derived seedlings.

#### 2.3.2. Identification of Positive Transgenic Seedlings

**Figure 3:**
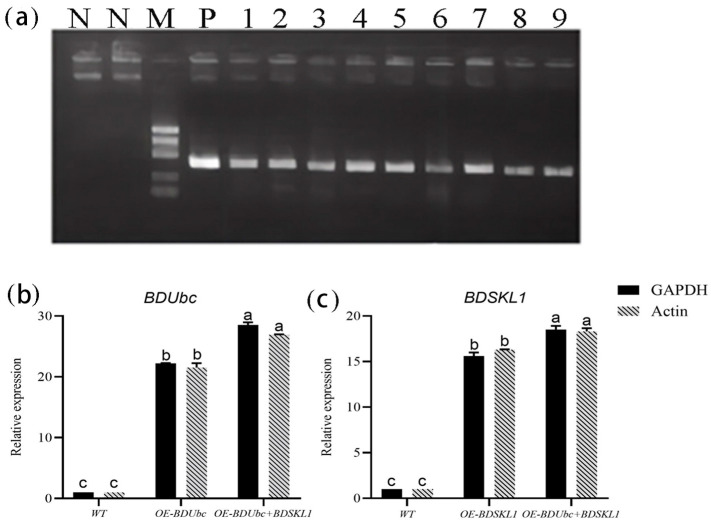
Validation of transgenic *B. pervariabilis* × *D. grandis* seedlings. (**a**) PCR validation of transgenic *B. pervariabilis* × *D. grandis* seedlings; M: 1000 DNA marker; N: negative control; P: positive control; 1, 2, 3: detection of the hyg501 fragment in OE-*BDUbc*-overexpressing plants; 4, 5, 6: detection of the hyg501 fragment in OE-*BDSKL1*-overexpressing plants; 7, 8, 9: detection of the hyg501 fragment in OE-*BDUbc* + *BDSKL1*-overexpressing plants; (**b**) relative expression levels of the *BDUbc* gene in transgenic *B. pervariabilis* × *D. grandis* seedlings; and (**c**) relative expression levels of the *BDSKL1* gene in transgenic *B. pervariabilis* × *D. grandis* seedlings. Different lowercase letters after the data in the same column indicate significant differences (*p* < 0.05) between treatments of different varieties after inoculation.

As shown in Figure 3a, the results indicate that the PCR analysis of the hygromycin fragment lengths matched the expected results, and sequencing validation confirmed their correctness. This suggests that positive plants were detected in OE-*BDUbc*, OE-*BDSKL1*, and OE-*BDUbc* + *BDSKL1*, indicating the successful transformation of *B. pervariabilis* × *D. grandis* by Agrobacterium. The transgenic differentiated seedlings can be used for further functional validation experiments. The transgenic lines overexpressing *BDUbc*, *BDSKL1*, and *BDUbc* + *BDSKL1* were named OE-*BDUbc*, OE-*BDSKL1*, and OE-*BDUbc* + *BDSKL1*, respectively. As shown in Figure 3b,c, the relative expression levels of the *BDUbc* gene in OE-*BDUbc* transgenic plants were significantly upregulated after transplantation, with values of 22.2 (*GAPDH*) and 21.5 (*Actin*), indicating the overexpression of the *BDUbc* gene in OE-*BDUbc* transgenic plants. Similarly, the relative expression levels of the *BDSKL1* gene in OE-*BDSKL1* transgenic plants were significantly upregulated after transplantation, with values of 15.6 (*GAPDH*) and 16.3 (*Actin*). In OE-*BDUbc* + *BDSKL1* transgenic plants, the relative expression levels of the *BDUbc* gene after transplantation were 28.5 (*GAPDH*) and 26.9 (*Actin*), while *BDSKL1* showed values of 18.5 (*GAPDH*) and 18.3 (*Actin*). This indicates that the expression levels of the *BDUbc* and *BDSKL1* genes in OE-*BDUbc* + *BDSKL1* transgenic plants were significantly higher compared to single overexpression of *BDUbc* or *BDSKL1* in *B. pervariabilis* × *D. grandis* transgenic plants.

### 2.4. Functional Validation of BDUbc and BDSKL1 in Transgenic Plants

#### 2.4.1. The Disease Resistance Level of Transgenic Plants

**Figure 4:**
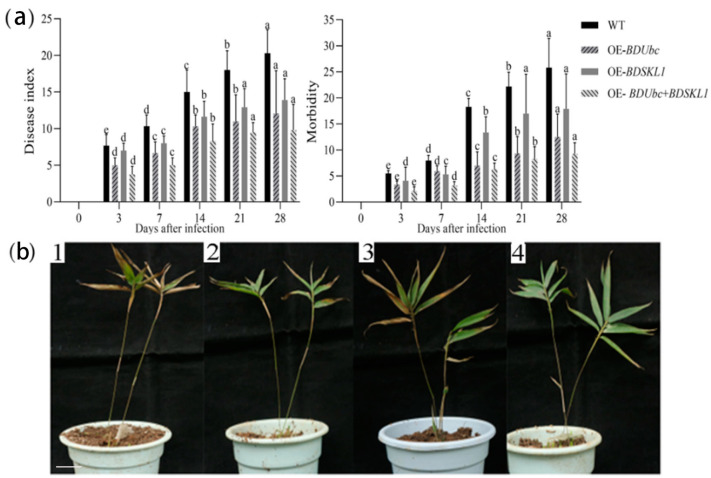
Disease resistance levels of transgenic plants. (**a**) Disease incidence and disease severity index of transgenic plants; different lowercase letters indicate significant differences (*p* < 0.05) among different varieties after inoculation; (**b**) symptom images of different strains after pathogen inoculation; 1: wild-type *B. pervariabilis* × *D. grandis*, 2: OE-*BDUbc*, 3: OE-*BDSKL1*, 4: OE-*BDUbc* + *BDSKL1*. One-year-old *B. pervariabilis* × *D. grandis* (40–50 cm plant height).

Using wild-type *B. pervariabilis* × *D. grandis* as a control, the statistical analysis revealed that the disease incidence rates of OE-*BDUbc*, OE-*BDSKL1*, and OE-*BDUbc* + *BDSKL1* were all significantly lower than those of wild-type *B. pervariabilis* × *D. grandis* (as shown in Figure 4a). Among them, the co-transformed plants, OE-*BDUbc* + *BDSKL1*, exhibited the lowest disease incidence rate and disease severity index (9.3 ± 2.1% and 9.8 ± 3.5, respectively) among the transgenic plants. OE-*BDUbc* transgenic plants had a disease incidence rate and disease severity index of 12.5 ± 4.4% and 12.1 ± 5.8, respectively, which ranked second after the co-transformed plants. OE-*BDSKL1* transgenic plants had a disease incidence rate and disease severity index of 17.9 ± 6.7% and 13.9 ± 2.9, respectively, making them the highest among the transgenic plants but still significantly lower than the values for wild-type *B. pervariabilis* × *D. grandis* plants.

As shown in Figure 4b, approximately 50% of wild-type *B. pervariabilis* × *D. grandis* leaves exhibited lesions 28 d after inoculation. Some lesions covered the entire leaf, resulting in the yellowing, wilting, and eventual shedding of the leaves, accompanied by branch dieback. In contrast, OE-*BDUbc*, OE-*BDSKL1*, and OE-*BDUbc* + *BDSKL1* transgenic plants showed only a few leaves with lesions, and the lesion areas were generally small. The entire plant did not exhibit branch dieback and remained healthy.

#### 2.4.2. Changes in BDUbc and BDSKL1 Expression in Transformed Plants during Different Periods of Infestation Detected by qRT-PCR

**Figure 5:**
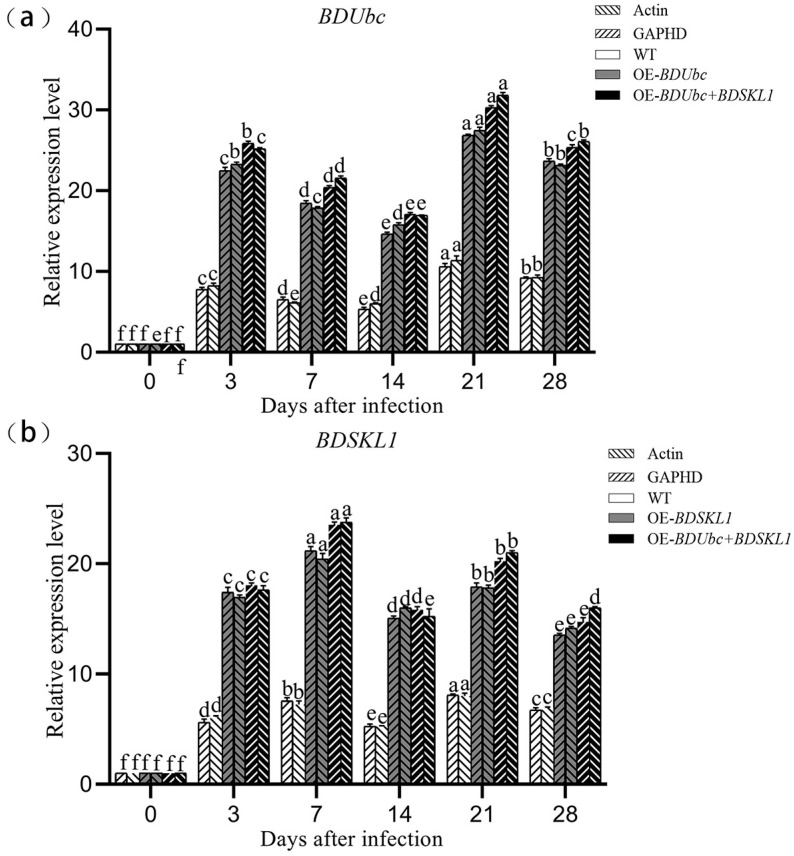
(**a**,**b**) Expression levels of *BDUbc* and *BDSKL1* genes at different infection time points in transgenic plants. Note: different lowercase letters indicate significant differences (*p* < 0.05).

As depicted in Figure 5, the results show that the expression levels of the *BDUbc* gene in transgenic plants, OE-*BDUbc* and OE-*BDUbc* + *BDSKL1*, significantly increased at 3 d post-inoculation, followed by a gradual decline, reaching their highest levels at 21 d post-inoculation before slowly decreasing again. In contrast, the expression levels of the *BDSKL1* gene in transgenic plants, OE-*BDSKL1* and OE-*BDUbc* + *BDSKL1*, significantly increased post-inoculation and reached their highest levels as early as 7 d post-inoculation. Subsequently, there was a gradual decline at 14 d, followed by another significant increase, a second peak at 21 d post-inoculation, and then a gradual decrease.

#### 2.4.3. Physiological and Biochemical Assays

**Figure 6:**
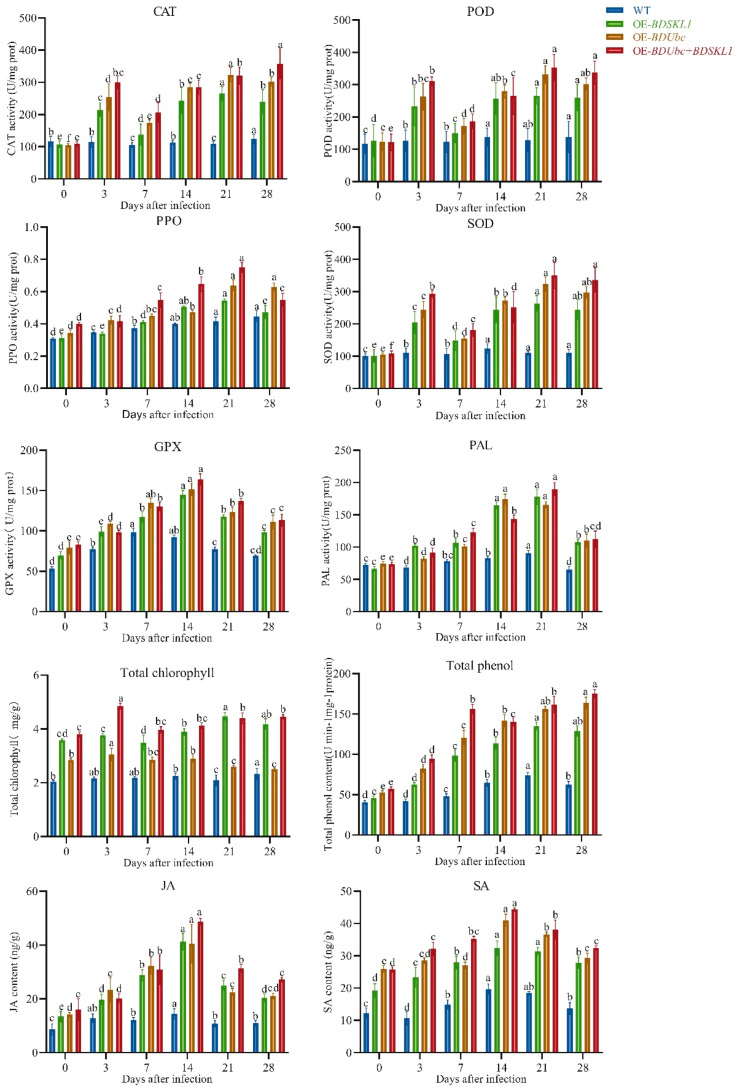
Changes in physiological and biochemical parameters in *B. pervariabilis* × *D. grandis* after infection. Note: different lowercase letters indicate significant differences (*p* < 0.05).

The physiological and biochemical assays were conducted using young shoots of *B. pervariabilis* × *D. grandis* inoculated with *A. phaeospermum* at 0 d, 3 d, 7 d, 14 d, 21 d, and 28 d. As shown in Figure 6, the results indicate that following the infection of *A. phaeospermum*, the levels of defense enzymes (CAT, PPO, POD, SOD, GPX, PAL), chlorophyll, total phenols, and plant hormones in the three transgenic lines (OE-*BDUbc*, OE-*BDSKL1*, and OE-*BDUbc* + *BDSKL1)* were higher compared to the wild-type plants. Among the defense enzymes (CAT, POD, and SOD), their trends were quite similar. Their levels rapidly increased at 3 d, followed by a gradual decline until reaching their peak levels at 21 d, after which they gradually decreased again. On the other hand, PPO, GPX, and PAL showed similar trends, initially increasing, reaching their peak values, and then decreasing. PPO and PAL reached their highest levels at 14 d, while GPX levels increased rapidly and peaked at 7 d. In OE-*BDUbc* transgenic plants, chlorophyll content was highest at 8 h post-infection and gradually decreased. All three transgenic lines (OE-*BDUbc*, OE-*BDSKL1*, and OE-*BDUbc* + *BDSKL1*) exhibited significantly higher levels of chlorophyll, total phenols, and plant hormones JA and SA compared to the wild-type. Chlorophyll content in transgenic plants increased slowly at 3 d, decreased at 7 d, and continued to rise thereafter. Total phenol content in all three transgenic lines showed a similar trend, rapidly increasing and reaching a peak at 7 d, followed by a gradual decline, with a subsequent increase beginning at 21 d and continuing to rise slowly. The levels of JA and SA increased rapidly after infection, and in all three transgenic lines, the highest levels of JA and SA were observed at 14 d, followed by a gradual decrease.

### 2.5. Subcellular Localization of BDUbc and BDSKL1

**Figure 7:**
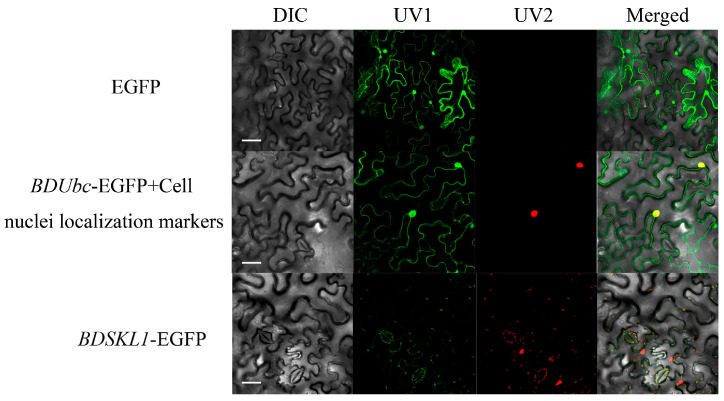
Subcellular localization of *BDUbc* and *BDSKL1*. Note: DIC, bright field; UV1, GFP fluorescence; UV2, RFP fluorescence, merged, superimposition of GFP fluorescence, RFP fluorescence and bright field. The white bar indicates 50 µm.

The results indicate that the positive control, transformed with the pSuper1300-GFP empty vector, displayed irregular and random fluorescence signals throughout the entire plant cell, confirming the success of the positive control setup. As shown in Figure 7, when the fusion plasmid pSuper1300-GFP-*BDUbc* and the nuclear marker were simultaneously introduced into tobacco, the *BDUbc* gene’s fluorescence signals overlapped with the nuclear marker signals and exhibited filamentous fluorescence signals at the cell periphery, indicating the subcellular localization of *BDUbc* in both the cell nucleus and the cytoplasm. In contrast, *BDSKL1*’s fluorescence signals coincided with the chloroplast’s intrinsic fluorescence, indicating the subcellular localization of *BDSKL1* in the chloroplast.

## 3. Discussion

In this study, the cloning sequences of the two target genes were found to exhibit a high degree of similarity with genes annotated as Ubc and SKL1 in the database. Furthermore, many of these genes were sourced from bamboo species, particularly those with the highest similarity to the sequences originating from bamboo. The analysis of conserved regions in the sequences revealed that both genes were highly conserved, with conserved motifs being identified across almost the entire sequence. Moreover, in the *BDUbc* gene, a ubiquitin-conjugating active-site signature and a ubiquitin-binding enzyme catalytic domain were identified. Subcellular localization revealed that *BDUbc* is located in the cell nucleus and cytoplasm, which was inconsistent with the initial prediction. However, this localization pattern aligns with previous findings in Arabidopsis [43], rice [44], and soybean [33] Ubc genes, which are known to be present in both the cell nucleus and cytoplasm. This consistency with previous research provides support for the experimental results. On the other hand, *BDSKL1* was found to localize in the chloroplast, which was consistent with the initial prediction. This localization pattern also agrees with the subcellular localization of SKL1 genes in *Arabidopsis* [45] and maize [39], as well as the experimental results obtained in this study.

The ubiquitin/proteasome system in plants plays a crucial role in growth, development, morphogenesis, and defense responses [25,46]. As early as 2006, it was discovered that the ubiquitin/proteasome system might play a significant role in the interaction between plants and microorganisms [47]. Ubiquitin-conjugating enzymes (E2) function as signal transduction factors in plant defense [48], and their roles have garnered increasing attention from researchers. Based on the results of qRT-PCR and physiological and biochemical analyses, we can speculate that the expression of different ubiquitin-conjugating enzyme genes may play a role in different defense response signaling pathways during *B. pervariabilis* × *D. grandis*’s interaction with *A. phaeospermum*. Ubiquitin-conjugating enzyme E2 (UBC) is a key enzyme in the ubiquitination process, playing a vital role in determining the length and topology of ubiquitin chains. Proteins labeled with K63-linked ubiquitin chains are primarily involved in signaling transduction [49]. Therefore, the early induced expression of ubiquitin-conjugating enzyme genes may be involved in the signal transduction of Phyllostachys edulis’ defense response. On the other hand, proteins marked with K48-linked ubiquitin chains are usually targeted for degradation by the 26S proteasome [49]. Hence, ubiquitin-conjugating enzyme genes induced later in the process may initiate defense responses in Phyllostachys edulis. For example, these genes can facilitate the degradation of some inhibitory proteins through the ubiquitination pathway, thereby initiating the defense response in *B. pervariabilis* × *D. grandis*.

The shikimate pathway plays a crucial role at the critical interface between primary and secondary metabolism, channeling carbon from glycolysis and pentose phosphate pathways towards synthesizing various physiologically important aromatic compounds [50]. It has been shown that the main role of SKL1 is to increase carbon fluxes from specific metabolite pools in response to environmental stresses or tissue-specific developmental requirements. qRT-PCR and physiological and biochemical results indicate that the shikimate kinase *BDSKL1* in response to *Alternaria brassicae* infection in *B. pervariabilis* × *D. grandis* gradually increases expression. Previous studies have shown that tomato SK, under induction by fungal elicitors, produces a direct response, potentially leading to the redirection of carbon flow toward the biosynthesis of plant toxins [51]. Therefore, during the early stages of pathogen invasion, *B. pervariabilis* × *D. grandis* increases photosynthesis and, through the SK pathway, directly induces the biosynthesis of toxins as a response. the downregulation of photosynthesis-related genes under stress conditions has previously been researched [52]. Similarly, the loss of SKL1-3 and SKL1-8 can lead to the loss of signals necessary for nuclear-encoded chloroplast development programs [50]. Hence, the gradual decrease in *BDSKL1* expression may be involved in suppressing the photosynthetic capacity of *B. pervariabilis* × *D. grandis* to cope with oxidative damage caused by reactive oxygen species produced during the infection process. Simultaneously, photosynthetic pigments may be degraded, possibly due to the increased activity of photosynthetic pigment-degrading enzymes or as a result of *A. phaeospermum* toxin production.

This study demonstrates that after infection with *A. phaeospermum*, the defense enzyme activity in the transgenic lines, OE-*BDUbc*, OE-*BDSKL1*, and OE-*BDUbc* + *BDSKL1,* is higher than in the wild-type lines, with OE-*BDUbc* + *BDSKL1* exhibiting the highest activity. Overall, defense enzyme activity shows an initial increase followed by a decreasing trend with prolonged infection time. Defense enzymes play a crucial role in plant disease resistance [53,54,55], indicating that pathogen infection activates the disease resistance mechanism in *B. pervariabilis* × *D. grandis*, increasing in defense enzyme activity. However, when the accumulation of toxic substances exceeds the clearing capacity of defense enzymes, defense enzyme activity decreases [56]. Upon infection, plants tend to increase their total phenolic content to inhibit the growth and reproduction of pathogens, and phenolic content is closely related to disease resistance [57], as is chlorophyll content [58,59]. Numerous studies have shown that higher chlorophyll content is associated with stronger plant disease resistance [60]. The transgenic lines significantly increased their total phenolic and chlorophyll content compared to the wild-type lines to resist pathogen infection [57,58,59,60]. Salicylic acid (SA) and jasmonic acid (JA) play crucial roles in plant–pathogen interactions, [61,62,63]. SA is pivotal in plant defense response signaling pathways and acquires resistance to such pathways [61]. After pathogen invasion, plants respond to the pathogen’s stress through the JA signaling pathway [64,65]. Following pathogen inoculation, the transgenic lines exhibited significantly higher JA and SA levels than the wild type, and OE-*BDUbc* + *BDSKL1* had the highest levels.

## 4. Materials and Methods

### 4.1. Materials

*A. phaeospermum* (stored in the Laboratory of Forest Protection Pathology, College of Forestry, Sichuan Agricultural University, Genbank accession number OK626768) and a *A. phaeospermum* spore suspension were used (conidia from PDA plates of *A. phaeospermum* spore Arthrospore cultured at 25 °C for 10 d were washed with sterile water and filtered, and the spore concentration was adjusted to 10^6^ cfu/mL). *Escherichia coli* DH5α, *Agrobacterium tumefaciens* GV3101 were purchased from Shanghai Ang Yu Biotechnology Co., Ltd. (Shanghai, China). One-year-old *B. pervariabilis* × *D. grandis* (40–50 cm plant height, ground diameter 1–1.5 cm); *B. pervariabilis ×D. grandis* seeds; 30 plants each from the transgenic lines, OE-*BDUbc*, OE-*BDSKL1*, OE-*BDUbc* + *BDSKL1*, and wild-type *B. pervariabilis* × *D. grandis* (wild-type *B. pervariabilis × D. grandis;* and seeds were purchased from Sichuan Biotechnology Innovation Science and Research Co. (Chengdu, China)). The expression vectors were pCAMBIA1301-35SN and pSuper1300-GFP (purchased from Wuhan Miao Ling Biotechnology Co., Ltd. (Wuhan, China)).

### 4.2. Methods

#### 4.2.1. Bioinformatics Analysis of BDUbc and BDSKL1

The parameters for constructing the phylogenetic tree were set as follows: The neighbor joining (NJ) method was employed using genetic distance [66] to construct the phylogenetic tree. A Bootstrap (value = 1000) correction was performed on the generated tree to generate the final phylogenetic tree. The conserved structural domains of the proteins were predicted using MEME (https://meme-suite.org/meme/tools/meme, 20 August 2023), and the important structural domains were predicted based on an E-value < 0.05. Phosphorylation sites were predicted using the NetPhos 3.1 Server (http://www.cbs.dtu.dk/services/NetPhos/, 20 August 2023). Subcellular localization predictions for the *BDUbc* and *BDSKL1* proteins were performed using the PSORT Prediction tool (https://www.predictprotein.org, 20 August 2023).

#### 4.2.2. Extraction of Total RNA and cDNA Synthesis in *B. pervariabilis* × *D. grandis*

Total RNA extraction from *B. pervariabilis* × *D. grandis* was performed using the TransZol RNA Extraction Kit (Beijing ComWin Biotech Co., Ltd., Beijing, China). The extracted products were assessed for purity and integrity using agarose gel electrophoresis (DYY-6D, Liuyi, Beijing, China) and a microvolume spectrophotometer (NANODROP, ThermoFisher Scientific CN, Shanghai, China). RNA samples meeting the desired criteria were reverse transcribed into cDNA using the All-in-One First-Strand cDNA Synthesis Super Mix for PCR (Beijing ComWin Biotech Co., Ltd., Beijing, China) following the manufacturer’s instructions, and the resulting cDNA was stored at −20 °C for further use.

#### 4.2.3. Cloning and Vector Construction of BDUbc and BDSKL1

Specific primer sequences were designed based on *BDUbc* and *BDSKL1* sequences from the NCBI database (Table S1). PCR amplification was performed using the cDNA of *B. pervariabilis* × *D. grandis* as a template. Homologous recombination primers for overexpression vectors, pCAMBIA1301-35SN-*BDUbc*, and pCAMBIA1301-35SN-*BDSKL1*, as well as subcellular localization vectors, pSuper1300-GFP-*BDUbc* and pSuper1300-GFP-*BDSKL1*, were also designed (Table S1). PCR reactions were conducted using cDNA as a template, and the PCR products were subsequently recovered. The pCAMBIA1301-35SN and pSuper1300-GFP vectors were digested using the restriction enzyme KpnI and incubated at 37 °C overnight. The digested fragments were purified and then ligated with the *BDUbc* and *BDSKL1* fragments, respectively, using the Trelief™ SoSoo Cloning Kit (Beijing TsingKe Biotech Co., Ltd., Beijing, China) at 50 °C for 15 min. The *BDUbc* was linked to the *Nos* terminator sequence, purified, and then combined with the linearized pCAMBIA1301-35SN vector using a homologous recombination to obtain the recombinant plasmid. Subsequently, the recombinant plasmid was digested, followed by the connection of the *Nos* terminator, *BDSKL1*, and the *MAS* promoter, resulting in the co-expression vector through homologous recombination. The ligated constructs were transformed into *E. coli* DH5α competent cells, and positive clones were identified using colony PCR. Plasmids from positive clones were extracted and sent for sequencing at Beijing TsingKe Biotech Co., Ltd. (Beijing, China) The sequences were compared to the correct sequences to confirm that the target genes had not undergone mutations, and the plasmids were prepared for further use.

#### 4.2.4. *A. tumefaciens* Mediated Cultivation of *B. pervariabilis* × *D. grandis* Transformants

The plant binary expression vectors pCAMBIA1301-35SN-*BDUbc* and pCAMBIA1301-35SN-*BDSKL1* were separately introduced into *A. tumefaciens* GV3101 (pSoup-19) competent cells using the freeze–thaw method [67], and positive clones were confirmed using colony PCR. Embryos of *B. pervariabilis* × *D. grandis* seeds were cross-sectioned [68], disinfected, and air-dried. The seeds were then inoculated onto MS medium supplemented with 500 mg/L proline, 500 mg/L glutamine, 300 mg/L casein hydrolysate, 30 g/L sucrose, 8 g/L agar, and 4 mg/L 2,4-D for callus induction under continuous light at 26 °C for approximately 20 days [1]. *A. tumefaciens* single colonies containing the desired vectors were cultured in media containing the corresponding antibiotics until the OD_600_ = 0.2. Subsequently, the bacterial cultures were co-cultured with the callus tissues [68]. Overexpression transformants were obtained after healing, screening, differentiation, and rooting.

#### 4.2.5. Verification of Transgenic Plants

The DNA of *B. pervariabilis* × *D. grandis* was extracted using EasyPure^®^ Plant Genomic DNA Kit (Beijing All Style Gold Biotechnology Co., Ltd., Beijing, China), and the obtained DNA was detected using the primer hyg501-F/R (Table S1), with *A. tumefaciens* as a positive control and ddH_2_O as a negative control, using a conventional PCR reaction system and sending the product to the company for sequencing. Using the transformed strain of bamboo tiliaceus, RNA was extracted and reverse transcribed into cDNA. qPCR was performed with *GAPDH* and *Actin* genes as internal references [2,69], the sequences of *BDUbc* and *BDSKL1* were referred to, and the primers for real-time fluorescence quantitative PCR were designed using Primier 5.0 with q*BDUbc*-F/R and q*BDSKL1*-F/R (Table S1). TransScript^®^ Green One-Step qRT-PCR SuperMix (TransGen, Beijing, China) was used for qRT-PCR. Three qPCRs were performed for each treatment group, the mean values were calculated, and the data were analyzed using the 2^−ΔΔCt^ method [70].

#### 4.2.6. Pathogen Infection and Pathogenicity Testing

After 8 weeks of transplantation, 30 uniform and vigorous young shoots of each variety of *B. pervariabilis* × *D. grandis* were selected. The spore suspension was inoculated into the nodes of the bamboo shoots using a needle puncture method [71], with four nodes per plant inoculated, and the inoculation was performed three times. Moist gauze was used for humidity control, and sterile water was used as a control. Disease assessment was conducted 28 d after infection, and disease incidence and severity index were calculated using the following methods: Disease Severity Index = [∑(Number of Plants at Each Disease Grade × Disease Grade)/(Total Number × Highest Disease Grade)] × 100 Disease Incidence (%) = Number of Diseased Plants/Total Number of Plants × 100. Disease assessment was conducted 28 d after infection, and disease incidence and severity index were calculated using Fang’s method [72].

#### 4.2.7. Changes in BDUbc and BDSKL1 Expression in Transformed Plants during Different Periods of Infestation, as Detected by qRT-PCR

The transformants were inoculated with *A. phaeospermum* at 0 d, 3 d, 7 d, 14 d, 21 d, and 28 d, and RNA was extracted and reverse transcribed into cDNA. q*BDUbc*-F/R and q*BDSKL1*-F/R were used as the primers for fluorescence quantitative PCR, and *GAPDH* and *Actin* genes were used as the internal references (Table S1). Three qPCRs were performed for each treatment group, the mean values were calculated, and the data were analyzed using the 2^−ΔΔCt^ method.

#### 4.2.8. Physiological and Biochemical Measurements

CAT activity was determined using the UV absorption method [73]. POD activity was measured using the guaiacol method [74]. PPO activity was determined using the pyrocatechol method [74]. SOD activity was assessed using the NBT assay [73]. GPX activity was measured according to the DTNB method [75]. PAL activity was determined using the UV spectrophotometric method [76]: weigh approximately 0.1 g of tissue and add it to 1 mL of extraction solution for cold homogenization. Subsequently, centrifuge the sample at 8000× *g*, 4 °C for 10 min, collect the supernatant, and keet it on ice for further analysis. Follow the instructions in the Solarbio kit manual (Beijing Solarbio Science & Technology Co., Ltd., Beijing, China). The calculation formulas are: CAT (U/mg prot) = [ΔA × V/(ε × d) × 10^6^]/(Vsample × Cpr)/T = 678 × ΔA/Cpr; POD (U/mg prot) = ΔA × V/(Vsample × Cpr)/0.01/T = 7133 × ΔA/Cpr; PPO (U/mg prot) = ΔA/0.01 × V/(Cpr × Vsample)/T = 60 × ΔA/Cpr; OD activity (U/mg prot) = [Percentage inhibition/(1 − Percentage inhibition) × V]/(Vsample × Cpr) × F = 11.11 × Percentage inhibition/(1 − Percentage inhibition)/Cpr × F; GPX (U/mg prot) = ΔAmeasurement/(ΔAstandard/Cstandard) × 1000 × Venzyme/(Cpr × Vsample)/T = 200 × ΔAmeasurement/ΔAstandard/Cpr; PAL (U/mg prot) = ΔA × V/0.1/(Cpr × Vsample)/T = 17.3 × ΔA/Cpr. Where V is the total reaction volume, ε is the molar absorption coefficient for H_2_O_2_, d is the cuvette’s path length, Vsample is the volume of the added sample, T is the reaction time, W is the sample mass, Cpr is the concentration of protein in the supernatant, and F is the sample dilution factor.

Chlorophyll content was measured using the ethanol extraction method [77]: Weigh approximately 0.1 g of tissue, homogenize it in 1 mL of distilled water, and then transfer it to a 10 mL test tube. Rinse the container with anhydrous ethanol and transfer all the washings to the test tube, adding enough anhydrous ethanol to reach 10 mL. Allow the mixture to soak in darkness or wrap in aluminum foil for 3 h until the residue turns white. The calculation formula is: Total Chlorophyll Content (mg/g) = (20.21 × A_645_ + 8.02 × A_663_) × Vextract × F ÷ W ÷ 1000 = 0.01 × (20.21 × A_645_ + 8.02 × A_663_) × F ÷ W. Where Vextract is the volume of the extraction solution, F is the dilution factor, and W is the sample mass.

Total phenolic content was determined using the Folin–Ciocalteu method [78]: Dry the sample until constant weight, grind, and sieve through a 30−50 mesh. Weigh about 0.1 g, add 2.5 mL of extraction solution, and perform ultrasonic extraction at 300 W and 60 °C for 30 min. Centrifuge at 12,000 rpm, 25 °C, for 10 min, collect the supernatant and adjust to 2.5 mL with extraction solution for testing. Follow the instructions provided by the Solarbio kit. The calculation formula is: Total Phenolic Content (mg/mg prot) = x × Vextract ÷ (Vextract × Cpr) = x ÷ Cpr. Where Vextract is the volume of the extraction solution, Cpr is the sample protein concentration, and x represents the measurement.

The activity of JA and SA was assessed using the ELISA method [79]: Take 0.1 g of fresh plant sample, grind it liquid nitrogen, and add nine times the volume of PBS buffer with a pH of 7.4. Centrifuge it at 4 °C for 30 min, and collect the supernatant for testing. Perform the determination according to the instructions provided by the Solarbio Jasmine Acid (JA) ELISA Kit and the Salicylic Acid (SA) ELISA Kit (Beijing, China). Calculate the quadratic regression equation of the standard curve using the concentration of the standard substance as the ordinate and the OD value as the abscissa. Substitute the sample’s OD value into this equation to calculate the sample concentration. The calculation method is: y = 37.878x^2^ + 738.88x − 24.694 (R^2^ = 0.9998).

#### 4.2.9. Subcellular Localization of BDUbc and BDSKL1

Tobacco grown at a temperature of 24 °C for approximately 30 d was selected for *A. tumefaciens* infiltration experiments. The pSuper1300-GFP-*BDUbc* and pSuper1300-GFP-*BDSKL1* constructs were separately introduced into *A. tumefaciens* GV3101 (pSoup-19) competent cells using the freeze–thaw method. Colonies were confirmed using PCR and grown in culture. A small incision was made on the underside of tobacco leaves using a needle, and the bacterial solution was injected into the leaf. The marked areas on the leaf were noted, and the tobacco leaves were incubated at around 21 °C for 2 d. Subsequently, the marked regions of the tobacco leaves were dissected and observed using a laser confocal microscope (FV3000, Olympus, Tokyo, Japan). GFP fluorescence was detected using a 495 nm excitation light and 507 nm emission filter, while RFP fluorescence was detected using a 532 nm excitation light and 588 nm emission filter for fluorescence imaging.

#### 4.2.10. Data Analysis

The data in this paper were plotted using GraphPad Prism 7 (GraphPad software Inc., La Jolla, CA, USA, 1989) and analyzed for significance using SPSS 27.0 (*p* < 0.05).

## 5. Conclusions

In summary, based on the analysis of disease incidence, the disease severity index, and physiological and biochemical parameters in transgenic lines, the results suggest that *BDUbc* and *BDSKL1* may act as disease-resistance genes in *B. pervariabilis* × *D. grandis* against blight. Studies have shown that the chloroplast-associated protein degradation pathway can regulate the transport of chloroplast proteins through the ubiquitin–proteasome system to alter chloroplast protein stability and mediate plant stress resistance [80]. Therefore, the *BDUbc* gene in *B. pervariabilis* × *D. grandis* may participate in resistance to blight by influencing changes in chlorophyll content mediated by *BDSKL1* through ubiquitination. This study provides evidence that *BDUbc* and *BDSKL1* may have a synergistic enhancing effect. Our existing research lacks an investigation of the upstream and downstream regulatory mechanisms of the *BDUbc* and *BDSKL1* genes. In our next research endeavors, we aim to explore the upstream and downstream regulatory genes associated with *BDUbc* and *BDSKL1*, along with their interacting proteins, validating the functions of these genes and proteins. We aim to construct a comprehensive disease-resistant regulatory network involving *BDUbc* and *BDSKL1*, delving deeper into novel transcriptional regulatory mechanisms in the resistance of *B. pervariabilis* × *D. grandis* to diseases. This endeavor aims to provide theoretical support for the future development of new strategies for preventing and controlling bamboo shoot blight.

## Data Availability

Data are contained within the article and supplementary materials.

## Supplementary Materials

The following supporting information can be downloaded at: https://www.mdpi.com/article/10.3390/ijms25010569/s1.
