# Supplementary material for: The Targeted Regulation of BDUbc and BDSKL1 Enhances Resistance to Blight in Bambusa pervariabilis × Dendrocalamopsis grandis
Source: Int J Mol Sci. 2024 Jan 1;25(1):569. doi: 10.3390/ijms25010569 (PMC10779405; doi:10.3390/ijms25010569)
Supplement: Supplementary file 1 [file ijms-25-00569-s001.zip › ijms-2726407-supplementary.pdf]

Table S1. Primers Used in This Study

Primer name	Primer sequence (5'-3')	Reference source
BDUbc-F	ATGGTGGACGTGTCGCGCGTGCAG	AGY80454.1
BDUbc-R	TCAGCCGGAGCAGAGCTTTTCAAG	AGY80454.1
BDSKL1-F	ACGGACTGCACTGCCAAGCGCAACAT	AIA26165.1
BDSKL1-R	CTAGAAGGGCCTTCCAGCAGCTTCCATC	AIA26165.1
q*BDUbc*-F	CTTGAAGACGGCGCTGCTAT	AGY80454.1
q*BDUbc*-R	GCACCTTTTCTTCCATGCCA	AGY80454.1
q*BDSKL1*-F	ATGTTCTTGGAGGCAAGGGT	AIA26165.1
q*BDSKL1*-R	TGAGTTCATGACGGCACCAT	AIA26165.1
GAPDH-F	GATTTTGTCGGTGATTCAAGGTC	Li et al., 2021
GAPDH-R	ATGTGAGCGATCAGATCCAGAA	Li et al., 2021
Actin-F	TGACAATGGCACTGGAATGG	Li et al., 2021
Actin-R	CCCATCCCTACCATGACACC	Li et al., 2021
hyg501-F	GAGCATATACGCCCCGGAGTC	Luo et al., 2022
hyg501-R	CAAGACCTGCCTGAAACCGA	Luo et al., 2022
pCAMBIA1301-35SN-*BDUbc*-F	ACCGCGGTGGAGCTCGGTACC ATGGTGGACGTGTCGCGCGTGCAG	AGY80454.1
pCAMBIA1301-35SN-*BDUbc*-R	CTCGAGGGGGGGCCCGGTACC TCAGCCGGAGCAGAGCTTTTCAAG	AGY80454.1
pCAMBIA1301-35SN-*BDSKL1*-F	ACCGCGGTGGAGCTCGGTACC ACGGACTGCACTGCCAAGCGCAACAT	AIA26165.1
pCAMBIA1301-35SN-*BDSKL1*-R	CTCGAGGGGGGGCCCGGTACCCTAGAAGGGCC TTCCAGCAGCTTCCATC	AIA26165.1
pSuper1300-GFP-*BDUbc*-F	TTAAATACTAGTGGATCCGGTACCATGGACTGC ACTGCCAAGCGCAACAT	AGY80454.1
pSuper1300-GFP-*BDUbc*-R	CTCGCCCTTGCTCACCATGGTACCGAAGGGCCT TCCAGCAGCTTCCATC	AGY80454.1
pSuper1300-GFP-*BDSKL1*-F	TTAAATACTAGTGGATCCGGTACCACGTGTCGC GCGTGCAG	AGY80454.1
pSuper1300-GFP-	CTCGCCCTTGCTCACCATGGTACCAAGGGCCTT	AGY80454.1

BDSKL1-R	CCAGCAGCTTCCATC

Note: The red part of the primers can be complementarily paired with sequences on the enzyme cleavage sites of the corresponding vectors, and the underline indicates the enzyme cleavage site. All primers were designed by PRIMER PREMIER 5.0 software. All sequences were obtained from whole genome and transcriptome databases. All accession numbers were obtained from GeneBank.

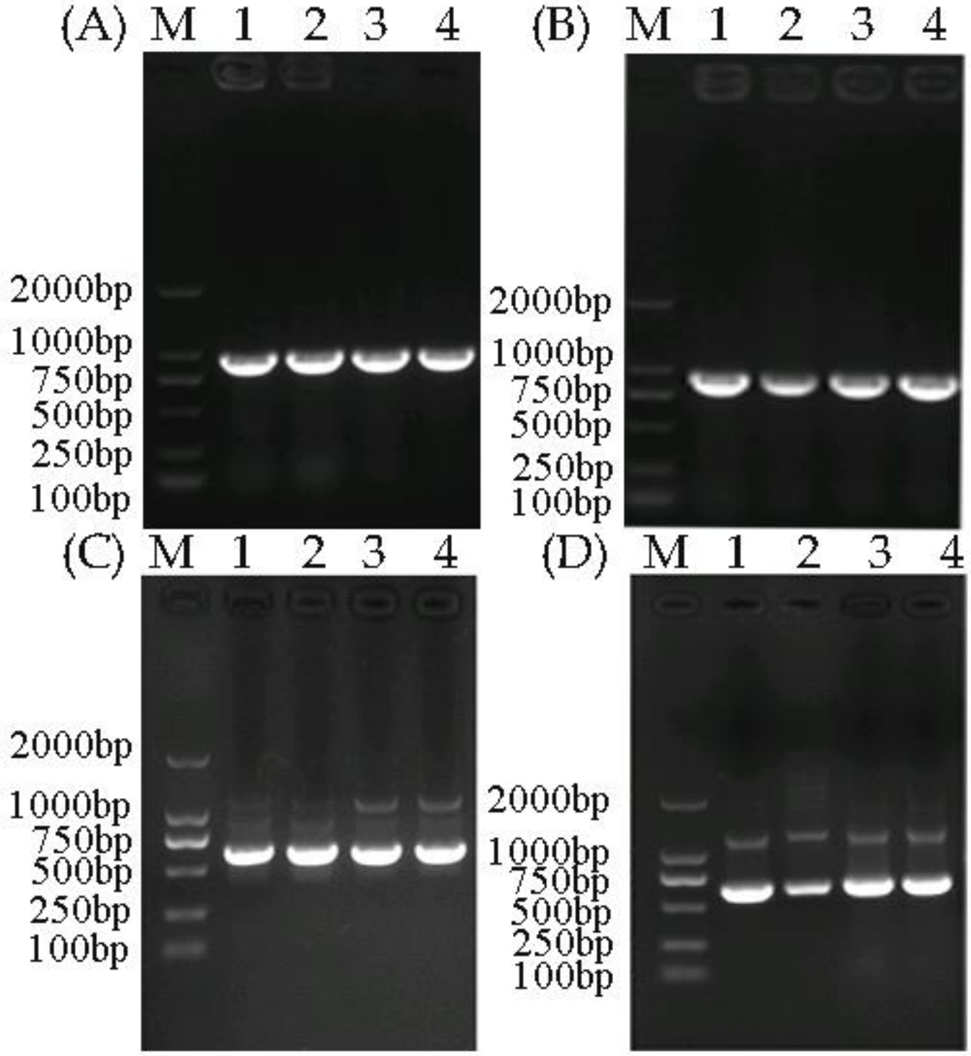

Figure S1. Colony PCR assay of vectors. (A) PCR assay of *E. coli* colonies with overexpression vector; (B) PCR assay of *A.tumefaciens* colonies with overexpression vector;(C) PCR assay of *E. coli* colonies with subcellularly localised vectors;(D) PCR assay of *A.tumefaciens* colonies with subcellularly localised vectors; 1, 2: *BDUbc*; 3, 4: *BDSKL1*.
